# *Escherichia coli* Occurrence and Antimicrobial Resistance in a Swine Slaughtering Process

**DOI:** 10.3390/pathogens13100912

**Published:** 2024-10-19

**Authors:** Aryele Nunes da Cruz Encide Sampaio, Evelyn Fernanda Flores Caron, Camila Koutsodontis Cerqueira-Cézar, Lára Cristina Bastos Juliano, Leonardo Ereno Tadielo, Patrícia Regina Lopes Melo, Janaína Prieto de Oliveira, José Carlos de Figueiredo Pantoja, Otávio Augusto Martins, Luís Augusto Nero, Fábio Sossai Possebon, Juliano Gonçalves Pereira

**Affiliations:** 1 School of Veterinary Medicine and Animal Science, São Paulo State University (UNESP), Botucatu Campus, Distrito de Rubião Jr, SN, Botucatu 18618-681, São Paulo, Braziljuliano.pereira@unesp.br (J.G.P.); 2 Food Inspection Laboratory, Department of Veterinary Medicine, Federal University of Viçosa, Viçosa 36570-900, Minas Gerais, Brazil; 3 Institute of Biotechnology (IBTEC), São Paulo State University (UNESP), Tecomarias av, SN, Botucatu 18607-440, São Paulo, Brazil

**Keywords:** enterobacteria, ESBL, MDR, pigs

## Abstract

The swine production chain can be a reservoir of antimicrobial-resistant *Escherichia coli*, which transfers resistance genes to other bacteria, serving as an important biomarker in the One Health approach. This study aimed to identify the frequency and antimicrobial resistance profile of *E. coli* in the swine production chain, assess the presence of extended-spectrum beta-lactamases (ESBL), and compare resistance profiles across different sample types. A total of 622 samples of swine carcasses from various points of the slaughter process (n = 400), swine feces (n = 100), commercial cuts (n = 45), environment (n = 67), and feces from employees (n = 10) of a pig slaughterhouse certified by the Federal Inspection Service, located in São Paulo state, Brazil, were collected. A total of 1260 *E. coli* isolates were obtained from the samples, with 73.6% of the samples testing positive. The agar disk diffusion test was performed with 10 different classes of antimicrobials. To confirm the production of ESBLs, the isolates were submitted to a double-disk synergism test using cefotaxime, ceftazidime, and amoxicillin with clavulanic acid. Of the total isolates, 80.71% were multidrug resistant. All ESBL-producing isolates were multidrug resistant and resistant to amoxicillin, tetracycline, and chloramphenicol. Isolates from human feces samples had less chance of being multidrug resistant than samples from other sources. The diversity of resistance profiles was verified in the samples, not clustering according to the sources, except for human feces isolates that clustered, evidencing lower antimicrobial resistance variability of these samples. Antimicrobial resistance is significantly present in the pork production chain, necessitating a comprehensive multidisciplinary approach to effectively mitigate risks within the One Health framework.

## 1. Introduction

To meet the growing demand for animal protein, the swine industry seeks higher productivity. Initially, subtherapeutic doses of antibiotics, called growth promoters, were used to modulate microorganisms in the gastrointestinal tract and improve production parameters, and soon, the use became continuous and intensified, surpassing prophylactic and therapeutic use. This selective pressure on microorganisms may have boosted the development of drug-resistant bacteria in pork products and other animal-origin food [1,2,3,4,5,6].

The swine production chain has also been mentioned as a potential reservoir of antimicrobial-resistant (AMR) *Escherichia coli*, due to strains that harbor mobile genetic material, such as plasmids, which can transfer these resistance genes to other *E. coli* strains or other species, in addition to the acquisition of genes from other microorganisms by conjugation. This makes these bacteria a biomarker for AMR, being an important microorganism in the context of One Health [7,8,9,10,11].

AMR is one of the biggest challenges for public health in the present and future, and must be recognized as an emerging global problem, which can affect human and animal health and impose social, economic, and environmental damages. So, a broad multidisciplinary approach is needed to reduce the risks to One Health [12,13]. This work aimed to characterize, compare the resistance profile, and confirm the production of extended-spectrum beta-lactamase enzymes (ESBL) from *E. coli* isolates from samples of humans, animals, pork products, and the environment of a swine slaughterhouse, contemplating a One Health approach.

## 2. Materials and Methods

### 2.1. Sampling

Samples were collected at a swine slaughterhouse located in São Paulo state, Brazil, with a capacity of 1500 animals per day and certified by the Federal Inspection Service. A total of 622 samples were collected from 10 batches raised in different farms. From each batch, animals (carcasses and feces), environment (equipment surface and residual and potable water), handlers with direct contact with the animals, and final products (commercial meat cuts) were sampled (Table 1).

The carcass samples, equipment surfaces, and final products were obtained using the swab technique with Nasco^®^ (Whirl-Pak, Madison, WI, USA) sponges previously hydrated with NaCl saline 0.85% (*m*/*v*). During sampling, sterile molds of 100 cm^2^ (10 cm × 10 cm) were used to delimit the area sampled. From each surface (internal and external carcasses, equipment, and final products) four distinct and representative points were evaluated, totaling 400 cm^2^ [14]. After collection, the sponges were placed in sterile containers and kept under refrigeration.

Animal feces were obtained using the rectal swab technique. These samples were stored in Cary Blair transport medium, refrigerated and sent to the laboratory for processing. The water samples were collected with a sterile flask containing sodium thiosulphate solution, under refrigeration, and sent to the laboratory for processing. 

The collection of human feces was carried out with the consent of the collaborators. Sterile containers, a swab in Cary Blair transport, and a manual collection with collection guidelines and recommendations were available. The feces were placed in a sterile vial, and the sample was transported to the tube containing the transport medium with a swab. These samples were identified and stored in isothermal boxes to be sent to the laboratory.

### 2.2. Escherichia coli Isolation 

Sponges from swab samples of carcasses, environment (equipment and utensils), and final products were processed adding 180 mL of buffered peptone water (APT, Oxoid Ltd., Basingstoke, Inglaterra) and homogenized in Stomacher at 185 rpm for 3 min and incubated at 37 °C for 18–24 h. Aliquots of the broth were streaked on MacConkey agar (MC, Oxoid^®^) and incubated at 37 °C for 18–24 h. From the water samples, 25 mL of the samples were aliquoted and 225 mL of APT were added, homogenized in Stomacher at 185 rpm for 3 min, and incubated at 37 °C for 18–24 h. Aliquots of the broth were streaked on MacConkey agar and incubated at 37 °C for 18–24 h. Swabs with samples of feces from animals and humans were plated directly on MacConkey agar and incubated for 18–24 h at 37 °C. The plates were examined, and five colonies with typical morphology for *E. coli* (pink colonies with bile precipitates) and two lactose non-fermenters of each morphological type were selected per sample. All selected suspicious colonies were streaked on tryptone soy agar (TSA, Oxoid Ltd., Basingstoke, Inglaterra) and incubated at 36 °C for 18–24 h. Confirmation was obtained using the IMViC test. Based on the biochemical characteristics of *E. coli*, the isolates were maintained in nutrient agar (Nutrient Agar, Oxoid Ltd., Basingstoke, Inglaterra) and brain–heart infusion broth (BHI, Oxoid Ltd., Basingstoke, Inglaterra) with glycerol. The isolates were kept in nutrient agar under refrigeration (4 °C) and frozen in BHI broth (−18 °C) for further analysis.

### 2.3. Antimicrobial Resistance Phenotypic Profile 

#### 2.3.1. Antimicrobial Sensitivity Profile by Disk Diffusion 

*E. coli* isolates were tested for resistance to 10 antimicrobial classes using the agar disk-diffusion method [15]. The choice of antimicrobials and concentrations were based on the Clinical and Laboratory Standards Institute(CLSI) [15] and Clinical and Laboratory Standards Institute Veterinary (CLSIVET) [16] to determine the sensitivity profile of enterobacteria: penicillins: amoxicillin (AMO) 10 µg; cephalosporins: ceftiofur (CTF) 30 µg, ceftazidime (CAZ) 10 µg, cefotaxime (CTX) 5 µg; carbapenems: imipenem (IPM) 10 µg; monobactams: aztreonam (ATM) 30 µg; aminoglycosides: gentamicin (GEN) 10 µg; tetracyclines: tetracycline (TET) 30 µg; quinolones: ciprofloxacin (CIP) 5 µg; folate inhibitors: sulfamethoxazole with trimethoprim (SUT) 23.75/1.25 µg; phenicols: chloramphenicol (CLO) 30 µg; and macrolides: azithromycin (AZI) 15 µg. Isolates were cultivated in BHI broth and incubated at 37 °C for 18–24 h. The cultures were diluted in sterile saline solution (NaCl 0.85%) until turbidity was compatible with the 0.5 degrees of the Mac Farland scale (approximately 1.5 × 10^8^ CFU/mL). The halo of inhibition was measured, and the result was previously classified as resistant, intermediate, or sensitive, based on the CSLI [15] and CLSIVET [16] recommendations. Control was carried out with a pan-susceptible strain of *E. coli* ATCC^®^ 25922. For all analyses (except for the dendrogram), isolates with an intermediate resistance profile were considered resistant. Strains with resistance to three or more classes simultaneously were considered multidrug resistant (MDR) [17].

#### 2.3.2. Detection and Confirmation of Extended-Spectrum Beta-Lactamase (ESBL) Producing *E. coli*

Following the EUCAST [18] screening methodology for ESBL enterobacteria, isolates that presented inhibition halos smaller than 22 mm for CAZ 10 µg and 21 mm for CTX 5 µg were submitted to the double-disc synergy test using third-generation cephalosporins (CTX and CAZ 30 µg) and an inhibitor of beta-lactamase (amoxicillin with clavulanic acid 20/10 µg). The control was carried out with a pan-susceptible strain of *Escherichia coli* ATCC^®^ 25922. Isolates that showed a widening of the inhibition halo of at least one cephalosporin towards amoxicillin with clavulanic acid were positive considerations for ESBL production.

### 2.4. Statistical Analysis

The percentages of positivity at each collection point, as well as resistance to the different antibiotics evaluated, occurrence of MDR, and ESBL were determined with Microsoft^®^ Excel^®^ 2019. The odds ratio of the isolate being MDR about the collection point was evaluated with the aid of Statistical Analysis Software—(SAS Institute, Cary, NC, USA). The resistant isolates with the most amount of antibiotics tested from each positive sample were selected to compose a dendrogram showing the distance of the different resistance profiles and indicating the different sampling groups, through the application Antibiotic Resistance Profiling of the Bionumerics software—version 8.1 using the Neighbor Joining method for clustering.

## 3. Results

### 3.1. Isolation of Escherichia coli

Of the 622 samples, 458 were positive for *E. coli*. Out of the 1260 isolates, 1178 were from animals (n = 258 after bleeding carcasses, n = 163 after scalding carcasses, n = 175 after evisceration carcasses, n = 165 after washing carcasses, and n = 346 feces), 71 from final products, 44 from equipment, and 41 from human feces. No isolates were obtained from water (Table 2).

The frequency of positive samples and isolates decreased through the slaughter process, with its peak in the animal feces and a decrease after bleeding and scalding, with a slight increase after the evisceration point and a further decrease in the final washing of pig carcasses point.

### 3.2. Antimicrobial Resistance Profile 

**Figure 1:**
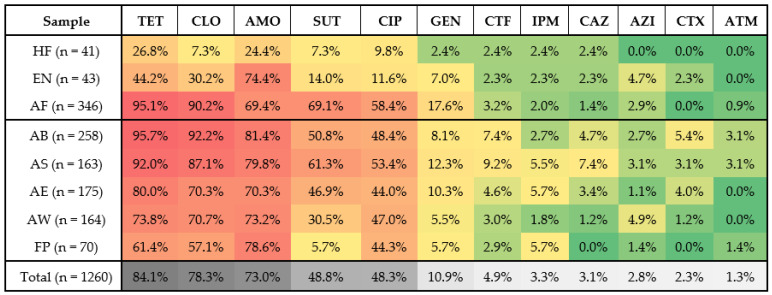
Percentage of resistance to different antibiotics of *Escherichia coli* isolates obtained from different collection points in a swine slaughterhouse in São Paulo state, Brazil. Red represents the highest percentages, green represents the lowest, and yellow represents the range between them. AB: after bleeding, AS: after scalding; AE: after evisceration; AW: after final washing; AF: animal feces; FP: final products; EQ: equipment; EN: environment; HF: human feces; AMO: amoxicillin; CTF: ceftiofur; CAZ: ceftazidime; CTX: cefotaxime; IPM: imipenem; ATM: aztreonam; GEN: gentamicin; TET: tetracycline; CIP: ciprofloxacin; SUT: sulfamethoxazole with trimethoprim; CLO: chloramphenicol; AZI: azithromycin.

High resistance to tetracycline (84.1%), chloramphenicol (78.3%), amoxicillin (73%), sulfamethoxazole with trimethoprim (48.8%), and ciprofloxacin (48.3%) were observed. However, for aztreonam (1.3%), imipenem (3.3%), cefotaxime (2.3%), ceftazidime (3.1%), and ceftiofur (4.9%), isolates were less resistant (Figure 1).

Isolates from human faces were resistant to tetracycline (26.8%), amoxicillin (24.4%), chloramphenicol (7.3%), sulfamethoxazole with trimethoprim (7.3%), ciprofloxacin (9.8%), lower resistance rates to gentamicin (2.4%), ceftiofur (2.4%), imipenem (2.4%), ceftazidime (2.4%), and sensitivity to other antimicrobials tested. The isolates from environmental samples showed a similar profile to animal samples, with a higher frequency of resistance to amoxicillin (74.4%), tetracycline (44.2%), and chloramphenicol (30.2%). The isolates obtained from animal carcasses, feces, and final products had high resistance to tetracycline (61 to 95.7%), chloramphenicol (57.1 to 92.2%), amoxicillin (69.4 to 81.4%), ciprofloxacin (44 to 58.4%), and sulfamethoxazole with trimethoprim (5.71 to 69.1%).

### 3.3. Multidrug Resistance Profiles

Out of the 1260 isolates, 88.66% (1117) showed multidrug resistance and only 8.01% (101) were susceptible to all drugs. Four classes of resistance were the most frequent (32.06%), followed by five (21.26%) and six classes (6.42%). Fewer than 1% were resistant to ten classes (0.15%), nine classes (0.23%), eight classes (0.39%), and seven classes (0.63%). MDR isolates were obtained from all collection points except water. There was a high frequency of multidrug resistance in isolates obtained from pigs after bleeding (93.79%), followed by isolates from swine feces (92.19%), carcasses after scalding (92.02%), carcasses after evisceration (73.14%), carcasses after the final wash (71.95%), and final products (54.92%). A total of 37.20% of the isolates from equipment surfaces (environment) were MDR. Contrary to what was observed in animal, environment, and food samples, *E. coli* isolates from human feces showed a lower frequency of MDR. In these samples, 12.19% of the isolates were resistant to three or more classes of antimicrobials (Table 2). A total of 112 MDR profiles were obtained, with 15 most prevalent (Table 3). There was a high frequency of five classes of antimicrobials represented (amoxicillin, gentamicin, tetracycline, ciprofloxacin, sulfamethoxazole with trimethoprim, and chloramphenicol), with emphasis on amoxicillin and tetracycline as the most frequent drugs. The AMO-TET-CIP-CLO profile was the most frequent, corresponding to 14.21%, present in samples from all origins and collected points (EN, AF, HF, AS, FP, AW, AB, AE).

Isolates from human feces had lower chances of being MDR when compared to isolates from other collection points, showing no statistical difference, except when compared to isolates obtained from environmental samples with 6.67 times more chances of being MDR (CI 0.78–50.00). The chances of isolates from animal fecal samples being MDR were 10 times higher when compared to environmental isolates (CI 3.70–25.00), and there were greater chances of being MDR when compared to isolates from different collection points such as after scalding (6.84), final products (5.40), after final wash (5.69), and after evisceration (4.43). Isolates from samples at the after-bleeding point were more likely to be MDR compared to isolates from after the scalding point (3.13), the final products (2.50), and after the final wash point (2.63). The other sample variables (samples × references) had no statistical differences (Table 4).

### 3.4. Comparison of Bacterial Resistance Profile to Antimicrobials at Different Collection Points

A dendrogram compares the resistance profile of the isolates. It was observed that the different sampled groups were distributed along the clusters, not evidencing a grouping in the resistance profiles regarding the origin of these samples, and showing the diversity of the resistance profiles. However, isolates from human feces groups are shown. Moreover, they showed less resistance compared to isolates from other sample sources (Figure S1—see Supplementary) 

### 3.5. Bacterial Resistance Profile of ESBL-Producing Strings

Out of the 1260 isolates, 20 (1.58%) were positive for ESBL production, of which 11 (55%) were from carcasses after bleeding, four (20%) from carcasses after scalding, three (15%) from carcasses after evisceration, and two (10%) from carcasses after final washing.

All 20 ESBL isolates had MDR profiles and were resistant to amoxicillin, tetracycline, and chloramphenicol. Only one isolate (5%) was not resistant to ceftiofur and sulfamethoxazole with trimethoprim. There was a high frequency of resistant isolates to ceftazidime (65%), cefotaxime (90%), and ciprofloxacin (60%). It was observed that only 3% of the isolates were resistant to imipenem, aztreonam, and azithromycin, and only one isolate (5%) was sensitive to gentamicin (Table 5).

## 4. Discussion

*E. coli* isolates were obtained from samples from all collected points except water. The highest percentages obtained, 93 and 90% of *E. coli* isolates from swine and human feces, respectively, are because *E. coli* is a commensal bacterium of the intestinal microbiota of warm-blooded animals [19,20,21,22]. The first collection point was after bleeding, and the isolation of *E. coli* can be associated to the previous stages of slaughter (transport and rest) since there is the possibility of contamination when lying on the ground and the fact that that the carcasses are all on the same conveyor belt without prior decontamination for each batch, which can lead to cross-contamination. 

Scalding consists of immersion of the carcasses in high-temperature water (62 to 63 °C) to achieve technical benefits when processing the carcasses [23,24]. Although this step is important for enterobacteria reduction [25], there were high positive frequencies at later sampling points. 

It was observed that after evisceration, the frequency of *E. coli* isolates was higher when compared to after scalding. This result can be related to cross-contamination of carcasses by contaminated equipment, recovery of non-cultivable cells, or technological processing failure in evisceration. Such facts can justify the positivity of samples collected from the surface of equipment and utensils, where more than half (57.4%) were positive for the presence of *E. coli*, which demonstrates to be an important source of cross-contamination for food [25,26,27]. Other authors also reported increased contamination after evisceration [28,29].

No significant decrease in contamination was observed even after the final wash. These results corroborate the study carried out in a swine slaughterhouse in Ireland, where there was an increase in contamination at this stage of the slaughter [30], which may have directly reflected in the contamination of the final products (71%) by *E. coli* strains.

Regarding the sensitivity profile of the isolates to antimicrobials, there was high general resistance to drugs used in human and veterinary medicine (tetracycline, amoxicillin, chloramphenicol, and sulfamethoxazole with trimethoprim), a worrying result when it is already known that the use of antimicrobials with similar active principles in humans and animals can lead to selection of the same resistance genes besides leading to lower drug efficacy, reduced antibiotic life, and longer hospitalizations [4,31,32]. Other studies with *E. coli* isolates from meat, swine, and human feces obtained similar sensitivity profiles to those in the present study [33,34]. Isolates from human feces also were resistant to antimicrobials commonly used in human and veterinary medicine. Less frequently, they show total sensitivity to the other seven antimicrobials tested, indicating that so far, they are not phenotypically resistant to so many classes of antimicrobials when compared to *E. coli* isolates from swine.

Isolates from environmental samples had similar sensitivity profiles to those from animal isolates, which may indicate that contamination continues during the production stages until the final product. When the carcass goes through the slaughter room, it can contaminate the entire line due to its direct contact with equipment, employees, and utensils [26]. Therefore, adequate sanitation is essential, as the greater the number of animals slaughtered per day, the greater the risk of cross-contamination.

The highest percentage of resistance was to tetracycline (83.57%), in agreement with the findings in the studies on swine production [35,36]. In a study in Denmark, where tetracycline is used mainly in pig farming, the tetracycline resistance profile was more pronounced among food isolates than in clinical samples [37]. Resistance to amoxicillin had the second highest frequency, accounting for 71.35% of the total isolates. A similar result was described in a study performed with *E. coli* isolates from humans, food, and water in Saudi Arabia, where 70.8% of the isolates were resistant to amoxicillin [33]. Resistance of the isolates to sulfamethoxazole with trimethoprim also occurred in most of the isolates (47.70%). The constant use of these antimicrobials in swine production, whose resistance gene is located in plasmids, which, in turn, encode resistance to tetracyclines, sulfonamides and penicillins [38], may justify the fact that these profiles are often found in *E. coli* isolates as well as in this study [33,35,38,39].

Although chloramphenicol for veterinary use was forbidden in Brazil [40], the presence of 76.59% of isolates resistant to this antibiotic is a concern since all isolates came from commercial farms. This might be explained by the maintenance through co-selection of resistance genes to this antibiotic with other resistance genes [41], and the fact that the use of florfenicol, a flowering derivative of chloramphenicol, is being increasingly reported, which may be contributing to the cross-resistance of the two drugs that frequently occur between antimicrobial agents belonging to the same class and acting by the same mechanism of action [42,43].

A high rate of MDR isolates was observed, similar to different authors [35,44]. These results can be associated with the large-scale use of antimicrobials in prevention and as growth promoters in industrial pig farming [45].

The appearance of *E. coli* MDR strains of different origins has been reported in several countries [46]. In this study, the most frequent profile (AMO-TET-CIP-CLO) occurred in all sampling points (Table 5). In Iceland, although antimicrobials are strictly prohibited as growth promoters, there are reports of MDR *E. coli* strains isolated from animals [47]. However, in this study, isolates from human feces had a low frequency of multidrug resistance when compared to other sample sources, corroborating with another study [47].

*E. coli* isolates had different resistance rates in different sampling points; there were different resistance profiles among the samples that were not clustered according to the sample origin, evidencing a diversity of resistance profiles. The fact that *E. coli* isolates from human feces (HF) were grouped into close clusters supports the results obtained, in which HF isolates are less resistant than isolates from other sources and with lower variability.

All 20 ESBL-producing isolates were resistant to more than five classes of MDR antimicrobials. There was a high frequency of resistance to drugs from the class of penicillins, cephalosporins, tetracyclines, quinolones, folate inhibitors, and phenolics. This result may rely on the fact that these enzymes are encoded by multi-resistance plasmids. Thus, ESBL-producing strains are mostly MDR [48]. No positive isolate for ESBL production came from a human fecal sample, a result similar to a study conducted in Tehran, Iran [49].

The limitation of this study could be that commensal *E. coli* were used instead of selecting pathogenic strains.

## 5. Conclusions

The study showed high general resistance of isolates to drugs used in human and veterinary medicine, multi-resistance in 80.71% of isolates, and ESBL-producing strains (1.58%). Isolates from human feces showed a lower frequency of antimicrobial resistance. The isolates had a diversity of resistance profiles in the sampling points, not clustering according to the origins of the samples, except for human feces isolates, evidencing the lower resistance and diversity of these strains. The results demonstrate the importance of the swine production chain as a reservoir of MDR strains.

## Figures and Tables

**Table 1:** Sample types, sampling points, and methods for *Escherichia coli* screening from a swine slaughterhouse in São Paulo state, Brazil.

Sample Types	Sampling Point	Method	Analytical Unit	Total
Animals	Carcasses—After bleeding	Swab	400 cm^2^	100
	Carcasses—After scalding	Swab	400 cm^2^	100
	Carcasses—After evisceration	Swab	400 cm^2^	100
	Carcasses—After the final wash	Swab	400 cm^2^	100
	Feces	Swab	Unity	100
Environment	Water (residual and potable)	Flask	100 mL	20
	Equipment and utensils surface	Swab	400 cm^2^	47
Humans	Employee feces	Swab	Unity	10
Food (final products)	Rump, rib, loin cup, shoulder, and shank	Swab	400 cm^2^	45
Total				622

**Table 2:** Number of samples, positive samples, *Escherichia coli* isolates, and multidrug-resistant isolates per collection point, obtained from a swine slaughterhouse in São Paulo state, Brazil.

Origin	Sampling Point	n of Samples	Positive Samples—n (%)	Isolates—n (%)	MDR—n (%)
Animals	AF	100	93 (93)	346 (27.46)	319 (92.19)
	AB	100	86 (86)	258 (20.47)	242 (93.79)
	AS	100	66 (66)	163 (12.93)	150 (92.02)
	AE	100	75 (75)	175 (13.88)	128 (73.14)
	AW	100	70 (70)	164 (13.01)	118 (71.95)
Pork products	FP	45	32 (71)	70	39 (54.92)
Environment	PW and RW	20	0 (0)	0 (0)	0 (0)
	EQ	47	27 (57.4)	43 (3.41)	16 (37.20)
Humans	HF	10	9 (90)	41 (3.25)	5 (12.19)
Total		622	458 (73.6)	1260 (100)	1017 (80.71)

AB: after bleeding, AS: after scalding, AE: after evisceration, AW: after final washing, AF: animal feces, FP: final products, PW: potable water, RW: residual water, EQ: equipment, HF: human feces.

**Table 3:** The most frequent MDR profiles among samples, the origin of *Escherichia coli* isolates, multidrug resistance, frequency and percentage frequency of the profiles.

Profile	Origins of Isolates	Frequency (%)
AMO-TET-CIP-CLO	EN, AF, HF, AS, FP, AW, AB, AE	179 (14.21)
AMO-TET-CIP-SUT-CLO	EN, AF, AS, AW, AB, AE	158 (12.54)
AMO-TET-CLO	EN, AF, HF, AS, FP, AW, AB, AE	129 (10.24)
AMO-TET-SUT-CLO	EN, AF, AS, AW, AB, AE	114 (9.05)
TET-CIP-SUT-CLO	EN, AF, AS, FP, AW, AB, AE	70 (5.56)
TET-SUT-CLO	AF, AS, AW, AB, AE	58 (4.60)
AMO-GEN-TET-CIP-SUT-CLO	EN, AF, AS, AW, AB, AE	47 (3.73)
AMO-GEN-TET-SUT-CLO	EN, AF, AS, AW, AB, AE	45 (3.57)
AMO-TET-CIP	AF, AS, AB, AE	18 (1.43)
TET-CIP-CLO	AF, AW, AB, FP	14 (1.11)
GEN-TET-SUT-CLO	AF, AS, AW, AB, AE	10 (0.79)

EN: environmental; AF: animal feces; AS: after scalding carcasses; AW: after final wash carcasses; AB: after bleeding carcasses; AE: after evisceration carcasses; FP: final products; HF: human feces; AMO: amoxicillin; CIP: ciprofloxacin; CLO: chloramphenicol; GEN: gentamicin; SUT: sulfamethoxazole with trimethoprim; TET: tetracycline.

**Table 4:** Calculation of odds ratio of isolates obtained from samples from different collection points to be MDR, concerning isolates from different points and their respective 95% confidence intervals.

Sample	Reference	Odds Ratio	Confidence Interval 95%		Statistical Difference
EN	AS	1.43	0.70	2.94	No
EN	FP	1.82	0.76	4.35	No
EN	AW	1.72	0.84	3.57	No
AS	FP	1.27	0.59	2.70	No
AS	AW	1.20	0.66	2.17	No
AS	AE	1.54	0.83	2.86	No
FP	AE	1.22	0.55	2.70	No
AW	AE	1.28	0.69	2.38	No
HF	AF	1.48	0.16	13.44	No
AB	AF	2.16	0.83	5.62	No
AS	HF	4.64	0.56	38.29	No
FP	HF	3.66	0.42	31.98	No
AW	HF	3.86	0.47	31.95	No
AB	HF	1.47	0.17	12.53	No
AW	FP	1.05	0.49	2.29	No
AE	AB	2.05	0.99	4.23	No
HF	AE	3.00	0.36	24.98	Yes
AF	EN	10.00	3.70	25.00	Yes
HF	EN	6.67	0.78	50.00	Yes
AB	EN	4.55	2.04	10.00	Yes
AE	EN	2.22	1.06	4.55	Yes
AB	AS	3.13	1.56	6.25	Yes
AB	FP	2.50	1.05	5.88	Yes
AB	AW	2.63	1.30	5.26	Yes
AF	AS	6.84	2.86	16.41	Yes
AF	FP	5.40	1.98	14.74	Yes
AF	AW	5.69	2.36	13.74	Yes
AF	AE	4.43	1.81	10.82	Yes

AS: after scalding carcasses; EN: environment; AF: animal feces; HF: human feces; FP: final products; AW: after final wash carcasses; AB: after bleeding carcasses; AE: after evisceration carcasses.

**Table 5:** Resistance profile of extended-spectrum beta-lactamase-producing *Escherichia coli* isolated from a swine slaughterhouse in São Paulo state, Brazil.

Isolate	Multiresistance Profile
1	AMO-CTZ-TET-CIP-CLO
2	AMO-CTF-CTZ-IPM-GEN-TET-CIP-SUT-CLO-AZI
3	AMO-CTF-CTZ-CTX-TET-CIP-SUT-CLO
4	AMO-CTF-CTX-TET-CIP-SUT-CLO
5	AMO-CTF-CTX-TET-CIP-SUT-CLO
6	AMO-CTF-CTZ-CTX-TET-CIP-SUT-CLO
7	AMO-CTF-CTZ-CTX-TET-CIP-SUT-CLO-AZI
8	AMO-CTF-CTX-TET-SUT-CLO
9	AMO-CTF-CTZ-CTX-TET-SUT-CLO
10	AMO-CTF-CTZ-CTX-IPM-ATM-TET-CIP-SUT-CLO
11	AMO-CTF-CTZ-CTX-TET-CIP-SUT-CLO
12	AMO-CTF-CTZ-CTX-ATM-TET-CIP-SUT-CLO
13	AMO-CTF-CTZ-CTX-TET-SUT-CLO
14	AMO-CTF-CTX-TET-SUT-CLO
15	AMO-CTF-CTZ-CTX-TET-SUT-CLO
16	AMO-CTF-CTX-TET-SUT-CLO
17	AMO-CTF-CTX-TET-SUT-CLO-AZI
18	AMO-CTF-CTZ-CTX-TET-CIP-SUT-CLO
19	AMO-CTF-CTZ-CTX-IPM-ATM-TET-CIP-SUT-CLO
20	AMO-CTF-CTX-TET-SUT-CLO

AMO: amoxicillin; ATM: aztreonam; AZI: azithromycin; CIP: ciprofloxacin; CLO: chloramphenicol; CTF: ceftiofur; CTX: cefotaxime; CTZ: ceftazidime; GEN: gentamicin; IPM: imipenem; SUT: sulfamethoxazole-trimethoprim; TET: tetracycline.

## Data Availability

The original contributions presented in this study are included in the article and Supplementary Materials. Further inquiries can be directed to the corresponding author.

## Supplementary Materials

The following supporting information can be downloaded at: https://www.mdpi.com/article/10.3390/pathogens13100912/s1, Figure S1: Dendrogram Obtained from the Comparison of the Profile of Bacterial Resistance to Antimicrobans of Escherichia coli Isolates Detailed.

## References

[1] Founou L.L., Founou R.C., Essack S.Y. (2016). Antibiotic Resistance in the Food Chain: A Developing Country-Perspective. Front. Microbiol..

[2] Aarestrup F.M. (2005). Veterinary Drug Usage and Antimicrobial Resistance in Bacteria of Animal Origin. Basic Clin. Pharmacol. Toxicol..

[3] Lancini J.B. (1994). Fatores exógenos na função gastrointestinal. Fisiologia da Digestão e Absorção das Aves.

[4] Moreno M.A., Domínguez L., Teshager T., Herrero I.A., Porrero M.C. (2000). Antibiotic Resistance Monitoring: The Spanish Programme. The VAV Network. Red de Vigilancia de Resistencias Antibióticas En Bacterias de Origen Veterinario. Int. J. Antimicrob. Agents.

[5] Scott A.M., Beller E., Glasziou P., Clark J., Ranakusuma R.W., Byambasuren O., Bakhit M., Page S.W., Trott D., Del Mar C. (2018). Is Antimicrobial Administration to Food Animals a Direct Threat to Human Health? A Rapid Systematic Review. Int. J. Antimicrob. Agents.

[6] Shobrak M.Y., Abo-amer A.E. (2014). Role of Wild Birds as Carriers of Multi-Drug Resistant *Escherichia coli* and *Escherichia vulneris*. Braz. J. Microbiol..

[7] Ewers C., Bethe A., Semmler T., Guenther S., Wieler L.H. (2012). Extended-Spectrum β-Lactamase-Producing and AmpC-Producing *Escherichia coli* from Livestock and Companion Animals, and Their Putative Impact on Public Health: A Global Perspective. Clin. Microbiol. Infect..

[8] Zheng H., Zeng Z., Chen S., Liu Y., Yao Q., Deng Y., Chen X., Lv L., Zhuo C., Chen Z. (2012). Prevalence and Characterisation of CTX-M β-Lactamases amongst *Escherichia coli* Isolates from Healthy Food Animals in China. Int. J. Antimicrob. Agents.

[9] Haenni M., Poirel L., Kieffer N., Châtre P., Saras E., Métayer V., Dumoulin R., Nordmann P., Madec J.Y. (2016). Co-Occurrence of Extended Spectrum β Lactamase and MCR-1 Encoding Genes on Plasmids. Lancet Infect. Dis..

[10] Barilli E., Vismarra A., Villa Z., Bonilauri P., Bacci C. (2019). ESβL *E. coli* Isolated in Pig’s Chain: Genetic Analysis Associated to the Phenotype and Biofilm Synthesis Evaluation. Int. J. Food Microbiol..

[11] Brisola M.C. (2018). Antimicrobianos utilizando a *Escherichia coli* como biomarcadora. Master’s Thesis.

[12] Queenan K., Häsler B., Rushton J. (2016). A One Health Approach to Antimicrobial Resistance Surveillance: Is There a Business Case for It?. Int. J. Antimicrob. Agents.

[13] Nguyen-Viet H., Chotinun S., Schelling E., Widyastuti W., Khong N.V., Kakkar M., Beeche A., Jing F., Khamlome B., Tum S. (2017). Reduction of Antimicrobial Use and Resistance Needs Sectoral-Collaborations with a One Health Approach: Perspectives from Asia. Int. J. Public Health.

[14] (2015). Microbiology of the Food Chain—Carcass Sampling for Microbiological Analysis.

[15] CLSI (2020). Performance Standards for Antimicrobial Susceptibility Testing.

[16] Clinical and Laboratory Standards Institute (2018). VET08 Performance Standards for Antimicrobial Disk.

[17] Magiorakos A.P., Srinivasan A., Carey R.B., Carmeli Y., Falagas M.E., Giske C.G., Harbarth S., Hindler J.F., Kahlmeter G., Olsson-Liljequist B. (2012). Multidrug-Resistant, Extensively Drug-Resistant and Pandrug-Resistant Bacteria: An International Expert Proposal for Interim Standard Definitions for Acquired Resistance. Clin. Microbiol. Infect..

[18] EUCAST (2017). EUCAST Guidelines for Detection of Resistance Mechanisms and Specific Resistances of Clinical and/or Epidemiological Importance.

[19] Tenaillon O., Skurnik D., Picard B., Denamur E. (2010). The Population Genetics of Commensal *Escherichia coli*. Nat. Rev. Microbiol..

[20] Brenner D.J., Krieg N.R., Staley J.R. (2005). Manual of Systematic Bacteriology.

[21] Feng P., Weagant S.D., Grant M.A., Burkhardt W. (2020). Enumeration of *Escherichia coli* and the Coliform Bacteria. Bacteriological Analytical Manual (BAM).

[22] Jay J.M. (2005). Microbiologia de Alimentos.

[23] MAPA—Ministério Da Agricultura, Pecuária e Abastecimento (1995). Portaria N° 711, de 1° de Novembro de 1995.

[24] Ministério DA Saúde (2018). Plano de Ação Nacional de Prevenção e Controle Da Resistência Aos Antimicrobianos No Âmbito Da Saúde Única 2018–2022.

[25] Bunci S., Sofos J. (2012). Interventions to Control Salmonella Contamination during Poultry, Cattle and Pig Slaughter. Food Res. Int..

[26] Terra N.N., Fries L.L.M. (2000). A Qualidade Da Carne Suína e Sua Industrialização. I Conferência Virtual Int. sobre Qual. Carne Suína.

[27] Ministério DA Saúde (2004). RESOLUÇÃO N° 216, DE 15 DE SETEMBRO DE 2004 Dispõe Sobre Regulamento Técnico de Boas Práticas Para Serviços de Alimentação.

[28] Arguello H., Carvajal A., Collazos J.A., García-Feliz C., Rubio P. (2012). Prevalence and Serovars of *Salmonella* Enterica on Pig Carcasses, Slaughtered Pigs and the Environment of Four Spanish Slaughterhouses. Food Res. Int..

[29] Seixas F.N., Tochetto R., Ferraz S.M. (2009). Presença de *Salmonella* sp. Em Carcaças Suínas Amostradas Em Diferentes Pontos Da Linha de Processamento. Ciência Anim. Bras..

[30] Mannion C., Fanning J., Mclernon J., Lendrum L., Gutierrez M., Duggan S., Egan J. (2012). The Role of Transport, Lairage and Slaughter Processes in the Dissemination of *Salmonella* spp. in Pigs in Ireland. Food Res. Int..

[31] OMS (2020). Resistência Antimicrobiana.

[32] van Breda L.K., Dhungyel O.P., Ward M.P. (2018). Antibiotic Resistant *Escherichia coli* in Southeastern Australian Pig Herds and Implications for Surveillance. Zoonoses Public Health.

[33] Aabed K., Moubayed N., Alzahrani S. (2021). Antimicrobial Resistance Patterns among Different *Escherichia coli* Isolates in the Kingdom of Saudi Arabia. Saudi J. Biol. Sci..

[34] Franco J.M.P.L., De Carvalho Mendes R., Cabral F.R.F., Menezes C.D.A. (2015). O Papel Do Farmacêutico Frente à Resistência Bacteriana Ocasionada Pelo Uso Irracional de Antimicrobianos. Sem. Acadêmica.

[35] Costa A.L.P. (2016). Resistência Bacteriana Aos Antibióticos: Uma Perspectiva Do Fenômeno Biológico, Suas Consequências e Estratégias de Contenção. UNIFAP.

[36] Franco R.M. (2002). *Escherichia coli*: Ocorrência Em Suínos Abatidos Na Grande Rio e Sua Viabilidade Experimental Em Lingüiça Frescal Tipo Toscana. Ph.D. Thesis.

[37] Jakobsen L., Spangholm D.J., Pedersen K., Jensen L.B., Emborg H.D., Agersø Y., Aarestrup F.M., Hammerum A.M., Frimodt-Møller N. (2010). Broiler Chickens, Broiler Chicken Meat, Pigs and Pork as Sources of ExPEC Related Virulence Genes and Resistance in *Escherichia coli* Isolates from Community-Dwelling Humans and UTI Patients. Int. J. Food Microbiol..

[38] Dunlop R.H., McEwen S.A., Meek A.H., Friendship R.M., Black W.D., Clarke R.C. (1999). Sampling Considerations for Herd-Level Measurement of Faecal *Escherichia coli* Antimicrobial Resistance in Finisher Pigs. Epidemiol. Infect..

[39] Brasilica A.V., Franco R.M., Pirola S., Mantilla S. (2010). Resistência antimicrobiana de *Escherichia coli* isoladas de carne e dejetos suínos. Acta Vet. Bras..

[40] MAPA—Ministério Da Agricultura, Pecuária e Abastecimento (2003). Instrução Normativa 9/2003.

[41] Rosengren L.B., Waldner C.L., Reid-Smith R.J. (2009). Associations between Antimicrobial Resistance Phenotypes, Antimicrobial Resistance Genes, and Virulence Genes of Fecal *Escherichia coli* Isolates from Healthy Grow-Finish Pigs. Appl. Environ. Microbiol..

[42] Arcangioli M.A., Leroy-Setrin S., Martel J.L., Chaslus-Dancla E. (2000). Evolution of Chloramphenicol Resistance, with Emergence of Cross-Resistance to Florfenicol, in Bovine *Salmonella* Typhimurium Strains Implicates Definitive Phage Type (DT) 104. J. Med. Microbiol..

[43] Bolton L.F., Kelley L.C., Lee M.D., Fedorka-Cray P.J., Maurer J.J. (1999). Detection of Multidrug-Resistant *Salmonella enterica* serotype typhimurium DT104 based on a gene which confers cross-resistance to florfenicol and chloramphenicol. J. Clin. Microbiol..

[44] Silva F.F.P., Santos M.A.A., Schmidt V. (2008). Resistência a Antimicrobianos de *Escherichia coli* Isolada de Dejetos Suínos Em Esterqueiras. Arq. Bras. Med. Vet. Zootec..

[45] Wang X.M., Jiang H.X., Liao X.P., Liu J.H., Zhang W.J., Zhang H., Jiang Z.G., Lü D.H., Xiang R., Liu Y.H. (2010). Antimicrobial Resistance, Virulence Genes, and Phylogenetic Background in *Escherichia coli* Isolates from Diseased Pigs. FEMS Microbiol. Lett..

[46] Sáenz Y., Briñas L., Domínguez E., Ruiz J., Zarazaga M., Vila J., Torres C. (2004). Mechanisms of Resistance in Multiple-Antibiotic-Resistant *Escherichia coli* Strains of Human, Animal, and Food Origins. Antimicrob. Agents Chemother..

[47] Thorsteinsdottir T.R., Haraldsson G., Fridriksdottir V., Kristinsson K.G., Gunnarsson E. (2010). Prevalence and Genetic Relatedness of Antimicrobial-Resistant *Escherichia coli* Isolated from Animals, Foods and Humans in Iceland. Zoonoses Public Health.

[48] Giuriatti J. (2017). Detecção De Beta-Lactamases De Espectro Estendido (Esbls) Em Isolados De *Salmonella* Provenientes De Carnes De Frango. Master’s Thesis.

[49] Doregiraee F., Alebouyeh M., Nayeri Fasaei B., Charkhkar S., Tajeddin E., Zali M.R. (2018). Changes in Antimicrobial Resistance Patterns and Dominance of Extended Spectrum β-Lactamase Genes among Faecal *Escherichia coli* Isolates from Broilers and Workers during Two Rearing Periods. Ital. J. Anim. Sci..

