# Supplementary material for: Escherichia coli Occurrence and Antimicrobial Resistance in a Swine Slaughtering Process
Source: Pathogens. 2024 Oct 19;13(10):912. doi: 10.3390/pathogens13100912 (PMC11510025; doi:10.3390/pathogens13100912)
Supplement: Supplementary file 1 [file pathogens-13-00912-s001.zip › pathogens-3251004-supplementary.pdf]

Figure S1 – DENDROGRAM OBTAINED FROM THE COMPARISON OF THE PROFILE OF BACTERIAL RESISTANCE TO ANTIMICROBANS OF *Escherichia coli* ISOLATES DETAILED

1 - Amoxicillin
2 - Ceftiofur
3 - Ceftazidime
4 - Cefotaxime
5 - Imipenem
6 - aztreonam
7 - Gentamicin
8 - Tetracycline
9 - Ciprofloxacin
10 - Sulfamethoxazole with trimethoprim
11 - Chloramphenicol
12 - Azithromycin

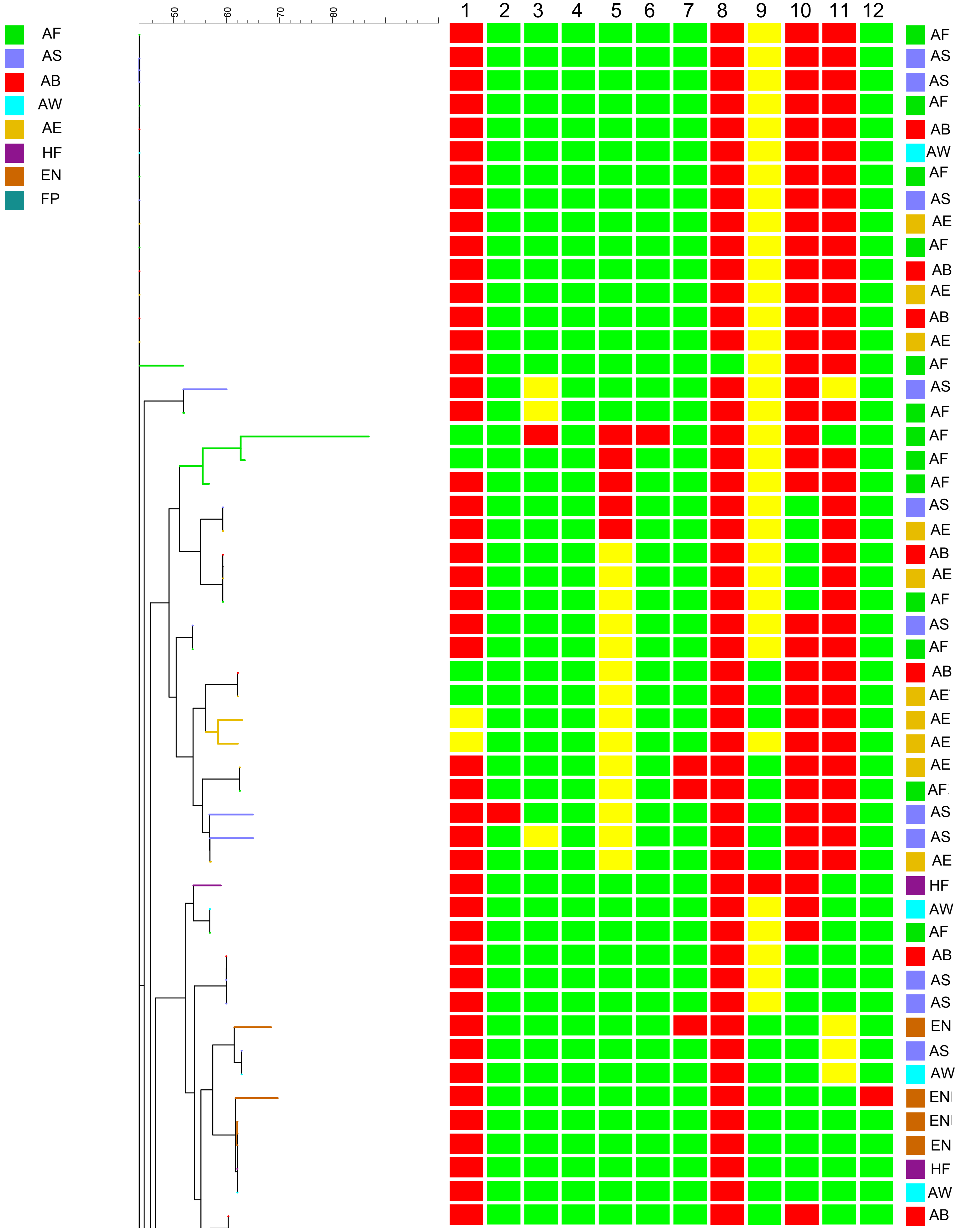

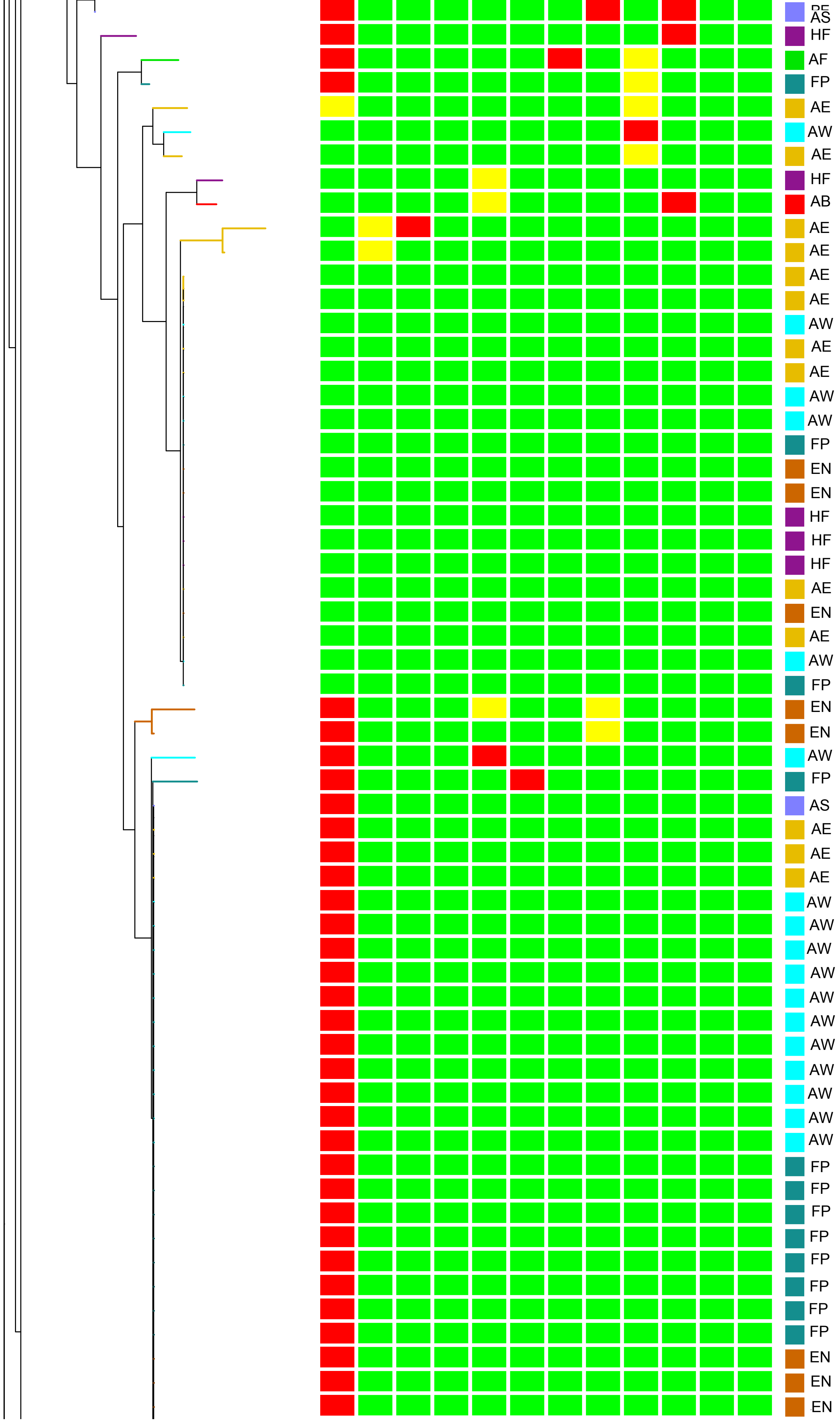
AS
HF
AF
FP
AE
AW
AE
HF
AB
AE
AE
AE
AE
AW
AE
AE
AW
AW
FP
EN
EN
HF
HF
HF
AE
EN
AE
AW
FP
EN
EN
AW
FP
AS
AE
AE
AE
AW
AW
AW
AW
AW
AW
AW
AW
AW
AW
FP
FP
FP
FP
FP
FP
FP
FP
EN
EN
EN

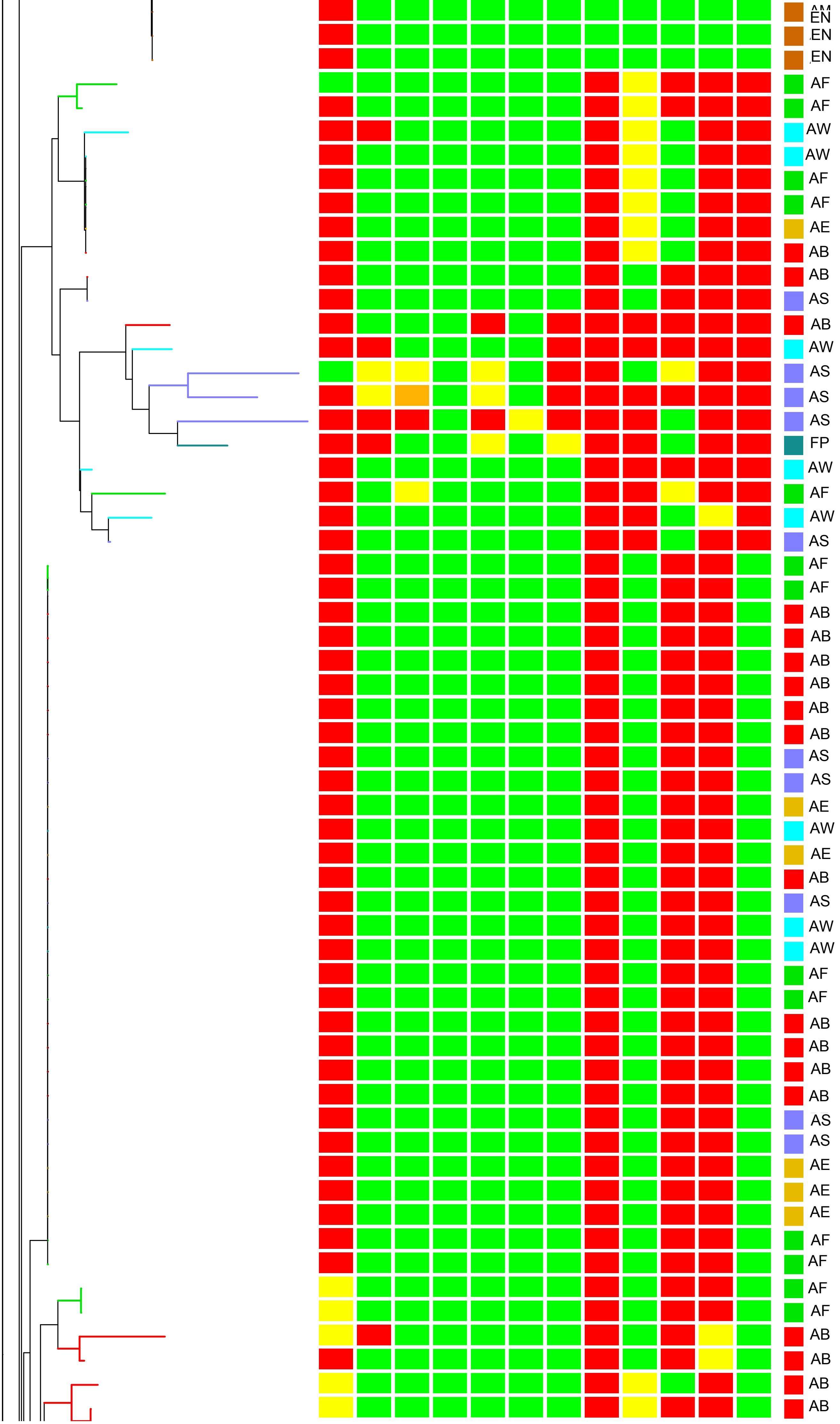
EN
EN
EN
AF
AF
AW
AW
AF
AF
AE
AB
AB
AS
AB
AW
AS
AS
AS
FP
AW
AF
AW
AS
AF
AF
AB
AB
AB
AB
AB
AB
AS
AS
AE
AW
AE
AB
AS
AW
AW
AF
AF
AB
AB
AB
AB
AS
AS
AE
AE
AE
AF
AF
AF
AF
AB
AB
AB
AB

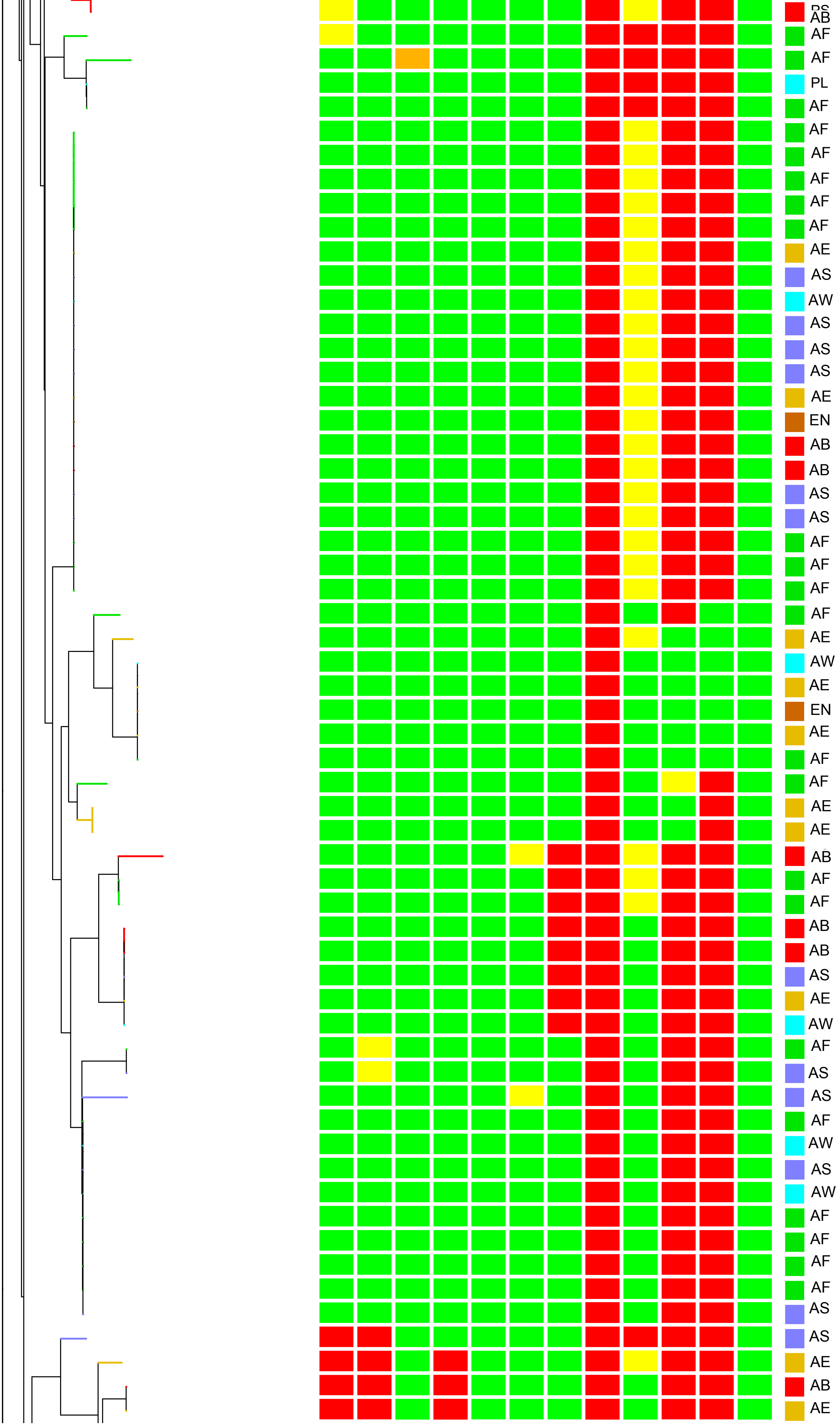
AB
AF
AF
PL
AF
AF
AF
AF
AF
AF
AE
AS
AW
AS
AS
AS
AE
EN
AB
AB
AS
AS
AF
AF
AF
AF
AE
AW
AE
EN
AE
AF
AF
AE
AE
AB
AF
AF
AB
AB
AS
AE
AW
AF
AS
AS
AF
AW
AS
AW
AF
AF
AF
AF
AS
AS
AE
AB
AE

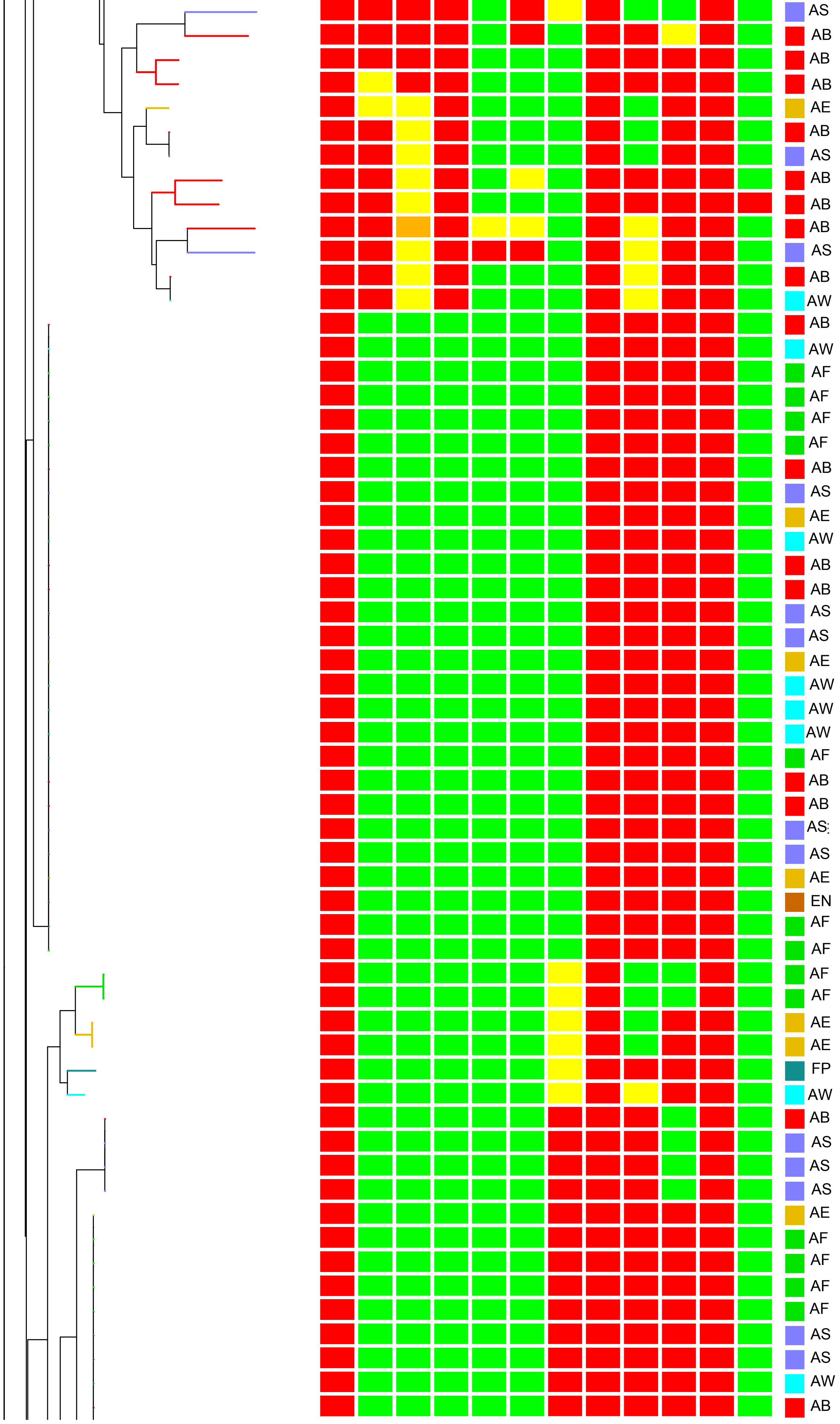
AS
AB
AB
AB
AE
AB
AS
AB
AB
AB
AS
AB
AW
AB
AW
AF
AF
AF
AF
AB
AS
AE
AW
AB
AB
AS
AS
AE
AW
AW
AW
AF
AB
AB
AS
AS
AE
EN
AF
AF
AF
AF
AE
AE
FP
AW
AB
AS
AS
AS
AE
AF
AF
AF
AF
AS
AS
AW
AB

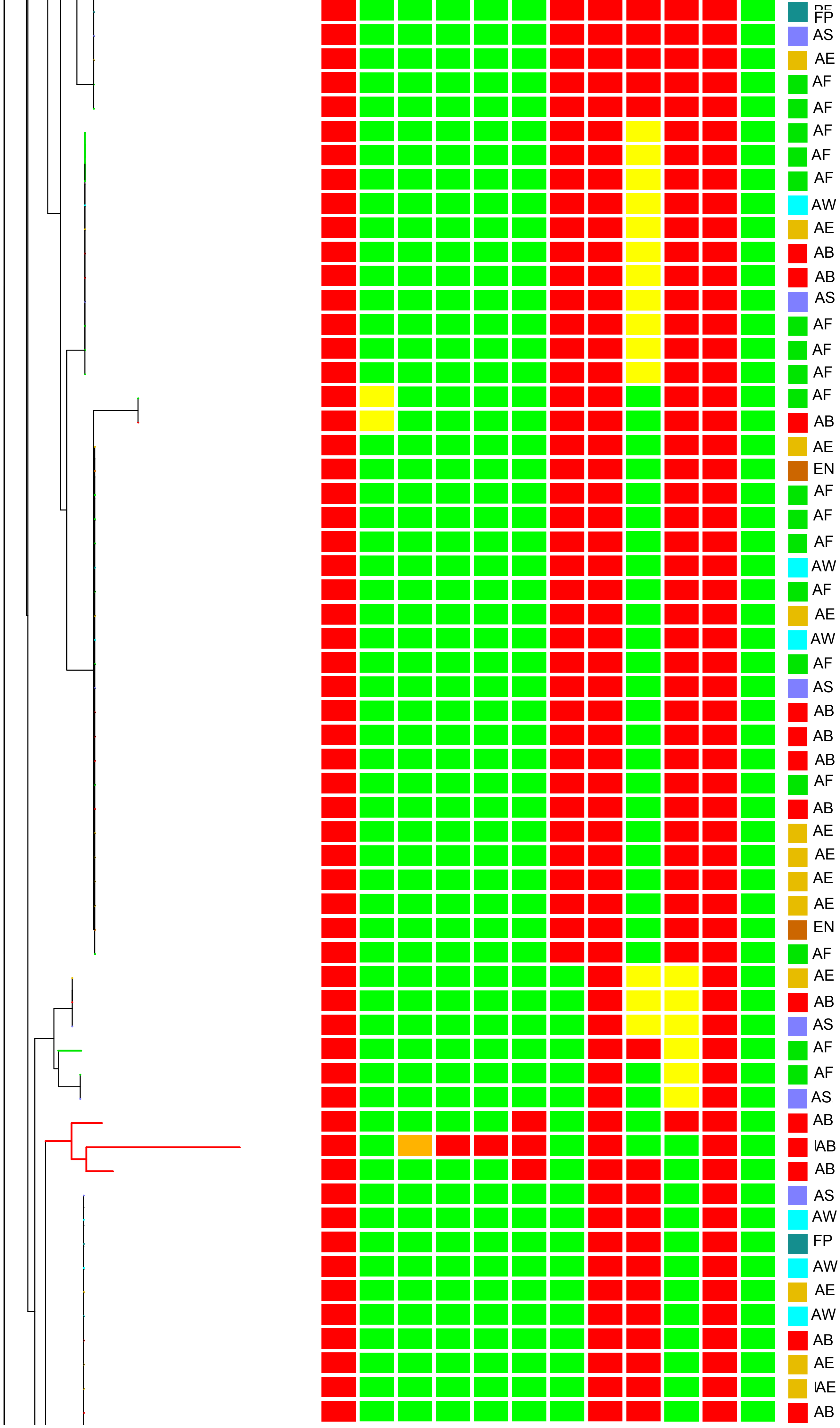
FP
AS
AE
AF
AF
AF
AF
AF
AW
AE
AB
AB
AS
AF
AF
AF
AF
AB
AE
EN
AF
AF
AF
AW
AF
AE
AW
AF
AS
AB
AB
AB
AF
AB
AE
AE
AE
AE
EN
AF
AE
AB
AS
AF
AF
AS
AB
AB
AB
AS
AW
FP
AW
AE
AW
AB
AE
AE
AB

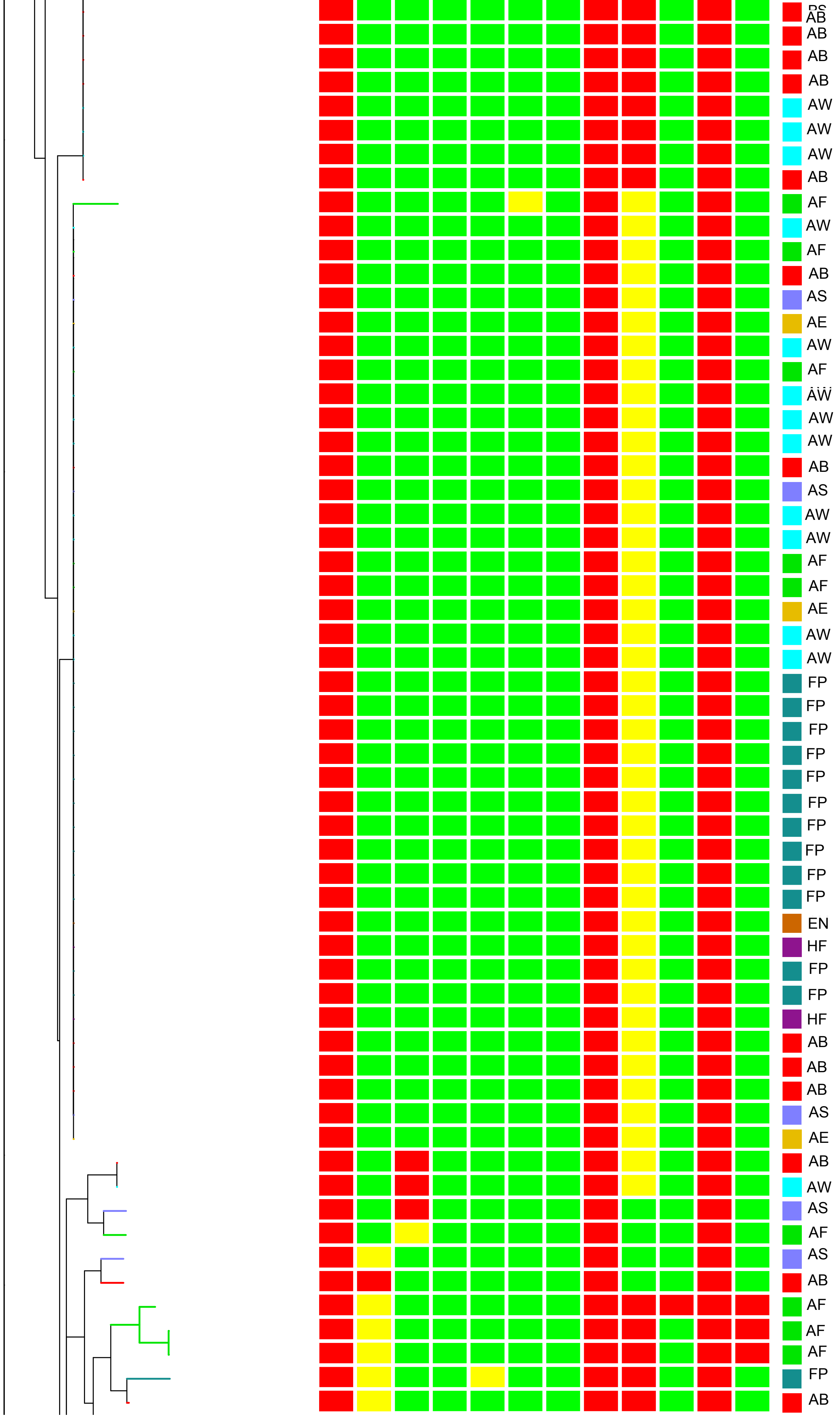
AB
AB
AB
AB
AW
AW
AW
AB
AF
AW
AF
AB
AS
AE
AW
AF
AW
AW
AW
AB
AS
AW
AW
AF
AF
AE
AW
AW
FP
FP
FP
FP
FP
FP
FP
FP
FP
FP
EN
HF
FP
FP
HF
AB
AB
AB
AS
AE
AB
AW
AS
AF
AS
AB
AF
AF
AF
FP
AB

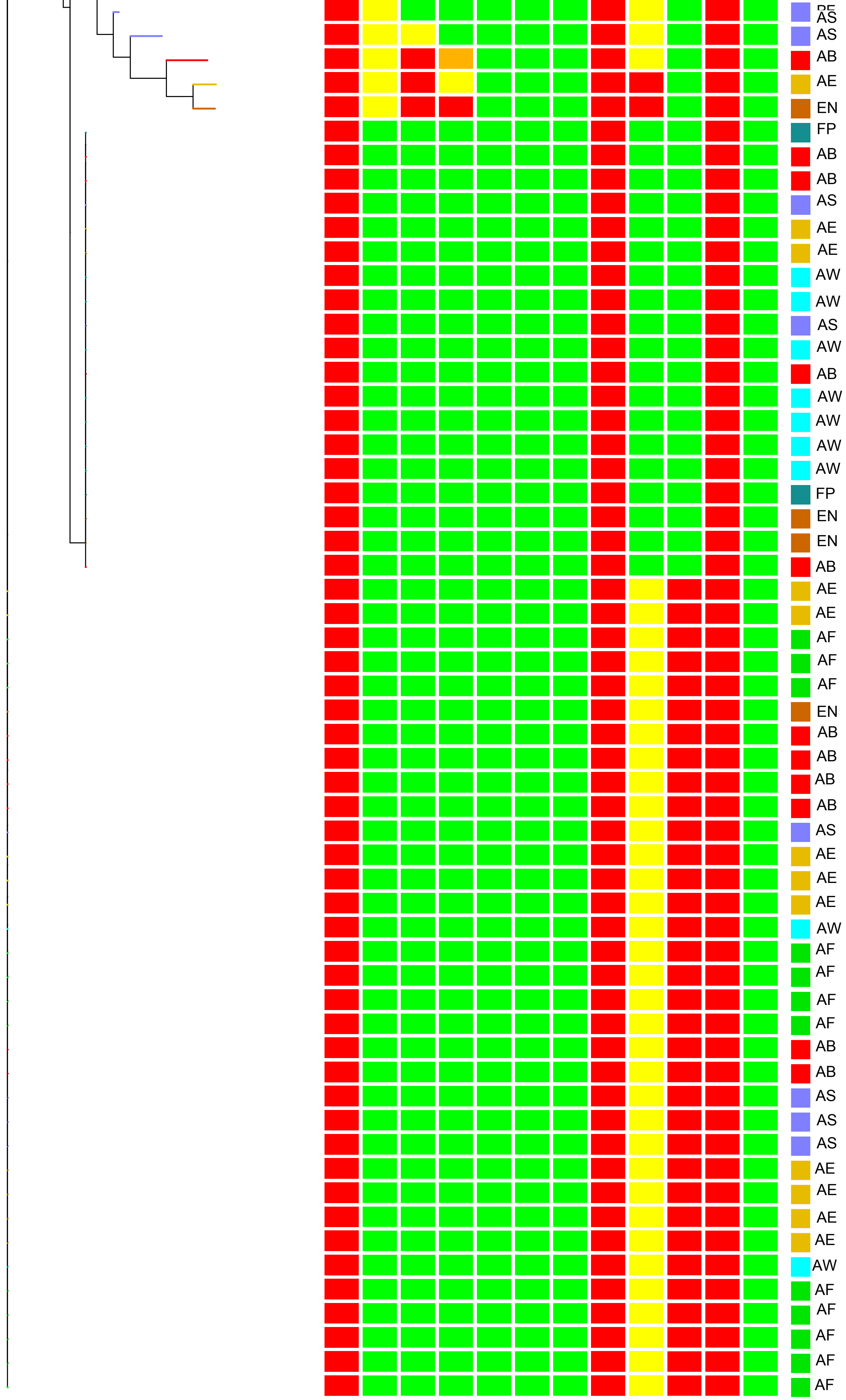
AS
AS
AB
AE
EN
FP
AB
AB
AS
AE
AE
AW
AW
AS
AW
AB
AW
AW
AW
AW
FP
EN
EN
AB
AE
AE
AF
AF
AF
EN
AB
AB
AB
AB
AS
AE
AE
AE
AW
AF
AF
AF
AF
AB
AB
AS
AS
AS
AE
AE
AE
AE
AW
AF
AF
AF
AF
AF
